# Coumarins as Fungal Metabolites with Potential Medicinal Properties

**DOI:** 10.3390/antibiotics11091156

**Published:** 2022-08-26

**Authors:** Olga M. Tsivileva, Oleg V. Koftin, Nina V. Evseeva

**Affiliations:** 1 Laboratory of Microbiology, Institute of Biochemistry and Physiology of Plants and Microorganisms, Saratov Scientific Centre of the Russian Academy of Sciences (IBPPM RAS), 13 Prospekt Entuziastov, Saratov 410049, Russia; 2 Department of Biochemistry, V.I. Razumovsky Saratov State Medical University, 112 ul. Bol’shaya Kazach’ya, Saratov 410012, Russia; 3 Laboratory of Immunochemistry, Institute of Biochemistry and Physiology of Plants and Microorganisms, Saratov Scientific Centre of the Russian Academy of Sciences (IBPPM RAS), 13 Prospekt Entuziastov, Saratov 410049, Russia

**Keywords:** natural compounds, coumarins, secondary metabolites, biosynthesis, fungi, endophytes, pathogenic bacteria, anticancer activity, antimicrobials, fungal biotechnology

## Abstract

Coumarins are a structurally varied set of 2*H*-chromen-2-one compounds categorized also as members of the benzopyrone group of secondary metabolites. Coumarin derivatives attract interest owing to their wide practical application and the unique reactivity of fused benzene and pyrone ring systems in molecular structure. Coumarins have their own specific fingerprints as antiviral, antimicrobial, antioxidant, anti-inflammatory, antiadipogenic, cytotoxic, apoptosis, antitumor, antitubercular, and cytotoxicity agents. Natural products have played an essential role in filling the pharmaceutical pipeline for thousands of years. Biological effects of natural coumarins have laid the basis of low-toxic and highly effective drugs. Presently, more than 1300 coumarins have been identified in plants, bacteria, and fungi. Fungi as cultivated microbes have provided many of the nature-inspired syntheses of chemically diverse drugs. Endophytic fungi bioactivities attract interest, with applications in fields as diverse as cancer and neuronal injury or degeneration, microbial and parasitic infections, and others. Fungal mycelia produce several classes of bioactive molecules, including a wide group of coumarins. Of promise are further studies of conditions and products of the natural and synthetic coumarins’ biotransformation by the fungal cultures, aimed at solving the urgent problem of searching for materials for biomedical engineering. The present review evaluates the fungal coumarins, their structure-related peculiarities, and their future therapeutic potential. Special emphasis has been placed on the coumarins successfully bioprospected from fungi, whereas an industry demand for the same coumarins earlier found in plants has faced hurdles. Considerable attention has also been paid to some aspects of the molecular mechanisms underlying the coumarins’ biological activity. The compounds are selected and grouped according to their cytotoxic, anticancer, antibacterial, antifungal, and miscellaneous effects.

## 1. Introduction

### 1.1. Overview on Fungal Coumarins

Natural compounds have provided the basic chemical foundation for manufacturing numerous biotechnologically important products and medicinal agents. Microorganisms present an essential source of natural products with various bioactive properties. Fungi are among the groups of microorganisms that are commonly recognized as the bioproducers of valuable metabolites and are being explored for compounds with medicinal applications. However, researchers have discovered only a small percentage of economically important metabolites, indicating future exciting products of fungal origin [1,2]. Novel natural products with varied chemical structures are constantly being reported from fungi, which are also an excellent source of coumarins. Coumarins (2*H*-chromen-2-ones) occupy a prominent place among natural products. These are benzopyrone compounds belonging to the flavonoid-like moiety of secondary metabolites. Opposite to a widely distributed opinion attributing coumarins to a group of mainly plant secondary metabolites, a large portion of the presently known coumarins exceeding 1300 [3,4] have been identified in bacteria and fungi [5,6]. Moreover, fungi occurred to be the most effective source of the coumarin-based products of pharmaceutical importance, never-before seen. The diversity of bioactivities among coumarins is so huge that the phrase “pharmacological promiscuity” has been applied in their case [3].

The natural products of coumarin nature discussed in this work are biosynthesized by fungi. Their bioactivities are of interest due to their unique structural features, providing applications in such fields of primary importance as cancer and neuronal injury or degeneration, microbial and parasitic infections, and others. As a result, fungal-sourced chemical agents of the coumarin class are of immediate interest to the pharmaceutical industry. This review is aimed at describing coumarins as one of several groups of biotechnologically valuable compounds typically derived from fungi. These microorganisms constitute a remarkably diverse, ubiquitous group of eukaryotes with the ability to synthesize many secondary metabolites [7], such as anticancer, antimicrobial, and antioxidant compounds. Fungi are capable of synthesizing products with the chemical structures of the important secondary metabolites from plants, including herbal coumarins, as well as novel substances with unpredictable architectures.

### 1.2. Novelty Statement

Recent exhaustive works comprise a concise summary of the latest knowledge of the biological and pharmaceutical properties of the plant-derived coumarins [8], emphasizing the plant–endophyte interaction as a key for the coumarins’ production as secondary metabolites [9]. The coumarin skeleton has attracted great interest in the development of new drugs. Cheke et al. (2022) [10] have reported on several methods for the synthesis of coumarin derivatives as antimicrobial agents. Stefanachi et al. (2018) [11] provided an overview of the most recent design and synthesis pathways leading to mono- and polyfunctionalized coumarins. The authors discussed multitarget coumarins addressing neurodegenerative diseases with a focus on inhibitors of monoamine oxidase, cholinesterase, and aromatase. Innovative synthetic methods have been described by Srikrishna et al. (2018) [12] for many molecules based on the coumarin ring system. These synthetic routes have led to interesting analogues of coumarins that possess pharmacological activities. The review by Zhu and Jiang (2018) [13] concluded that the specific site on the core structure of coumarin exhibits one or more activities due to the electronic or steric effects of the substituents. This review is intended to be conducive to rational design and development of more active and less toxic agents with a coumarin scaffold. In the review by Hu et al. (2017) [14], coumarin and its derivatives as anti-tuberculosis agents have been reviewed and outlined the critical aspects of design and structure-activity relationship of these coumarin derivatives. In addition, Fan et al. (2018) [15] reviewed several fluoroquinolone hybrids, including fluoroquinolone-coumarin hybrids, as anti-tuberculosis agents.

A rather large number of recent reviews concerned with coumarins prompted us to make drastic selections. Considering this in mind, the present review evaluates coumarins the fungal-sourced bioactive agents, their structure-related peculiarities, and their future therapeutic potential. Special attention has been paid to the fungal-derived biological activities, mainly cytotoxic, antibacterial, antitubercular, antifungal, and antiviral natural coumarins, as well as other coumarins of pharmacological value from fungi. A particular emphasis has been placed on the coumarins, successfully bioprospected from fungi but originally known as exclusively herbal metabolites. This review also highlights some aspects on molecular mechanisms underlying the coumarins’ biological activity. The coumarin derivatives studied in the course of clinical and preclinical testing have also been taken into account, but not in an extensive mode, most of these coumarins being not fungal-derived but (semi)-synthetic hybrid compounds and being already extensively quoted in the comprehensive reviews characterized above.

## 2. Coumarins Hold a Special Place among Natural Products

**Figure 1:**
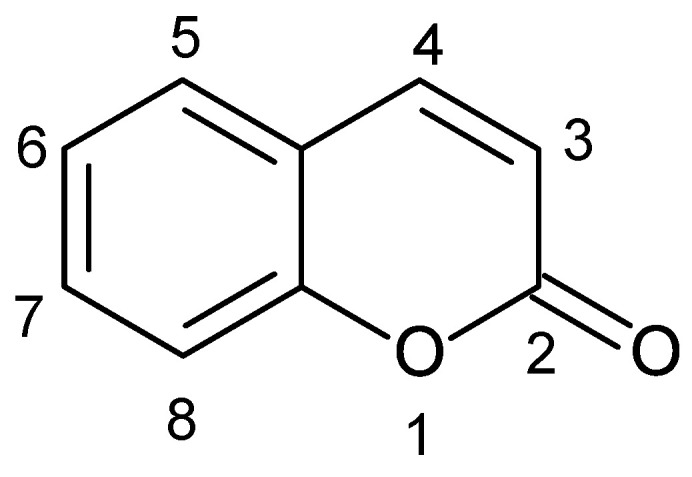
Structural formula of coumarin (2*H*-1-benzopyran-2-one) with atom numbering.

Coumarin (2*H*-1-benzopyran-2-one, or 2*H*-chromen-2-one) (Figure 1) is a parent compound of a class of coumarins, which are lactones consisting of a benzene ring fused to an alpha-pyrone ring.

A key bicyclic heterocycle, 2H-chromen-2-one, is found to be a natural secondary metabolite extracted from plants and microorganisms. The name “coumarin” derives from the French term “coumarou” that refers to *Dipteryx odorata* (*Coumarouna odorata*) seeds, one of the sources from which coumarins were first isolated. Those benzopyrone derivatives belong to flavonoid-like groups of secondary metabolites, first from plants. Diverse families of plants have been used to isolate coumarins [16]. A few years ago, it was reported that more than 1300 coumarins have been identified in plants, bacteria, and fungi [17]. Coumarins (2*H*-chromen-2-ones) occupy a prominent place among natural products.

Natural products have been playing a major role in the search for novel drugs for numerous illnesses. Prominent producers of these natural products are found in fungi [18]. One should remember that the discovery of the antibiotic penicillin from a fungus almost a century ago heralded an era of intense exploration of compounds from prokaryotic and eukaryotic organisms for their pharmaceutical potential. It was the tremendous success of penicillin as an antibiotic in the early 1940s that shifted the focus of natural product-based drug sources from plants to microorganisms [1]. Throughout human history and especially in the past century, natural products have remained virtually undisputed leaders among the various therapeutic tools humans have employed to combat numerous diseases, including cancer.

Nevertheless, plant sources’ utilization for obtaining these valuable molecules, secondary metabolites with anti-inflammatory, anticoagulant, antibacterial, antifungal, antiviral, anticancer, antihypertensive, antitubercular, anticonvulsant, antiadipogenic, antihyperglycemic, antioxidant, and neuroprotective properties [19] presents many formidable challenges towards their reliable supply. Because of seasonal variations and large-scale deforestation concerns in plants, the alternative sources of microbial nature have attracted considerable interest among academic researchers and commercial representatives. While plant-originating products have been playing an important role in traditional medicine for thousands of years, more or less comprehensive research into fungal natural products became possible with the development of natural sciences during the 20th century. The secondary metabolites of microbial origin are well known as a valuable supply of lead molecules in the selection of drug candidates against infectious diseases, cancer, and many other illnesses. Endophytic microorganism, especially fungi, are potential sources of known and novel natural products. The ever-increasing demand of potent drugs in the face of a less successful traditional plant-based industrial pipeline has virtually brought these enigmatic microorganisms to unprecedented biomedical prominence.

Coumarins, which are heterocyclic oxygenated substances found in plants, have been found in many families, including Rutaceae, Umbelliferae, Asteraceae, Fabaceae, Rosaceae, Solanaceae, and Moraceae, with approximately 200 coumarins identified in Rutaceae alone [20]. Since the first isolation of coumarins as a natural product from plants, they have been routinely implemented as herbal remedies starting from the onset of herbal medicine [21]. Coumarins are one of several groups of medicinally important compounds derived from fungal endophytes in plants, which also include alkaloids, flavonoids, glycosides, lignans, phenylpropanoids, quinones, saponins, terpenoids, xanthones, and other compounds. More complex coumarin derivatives, coumarin-fused heterocyclic compounds, have exhibited intriguing physiochemical properties and biological and pharmaceutical activities [22]. Coumarins have been investigated as one of the promising structures for the development of novel agents with higher specificity and affinity to molecular targets, and are characterized by their intrinsic properties as antimicrobial [23], antioxidant, anti-inflammatory [24,25,26], antiadipogenic [27], cytotoxic [28], apoptosis [29], antiproliferative [30], antimycobacterial activities against *Mycobacterium tuberculosis* [31], antileishmanial [32], antiviral [16,33], anticancer [34], and cytotoxic [35] agents. Due to this wide range of pharmacological values, coumarins and their derivatives have more importance in synthesis and production.

## 3. Herbal Coumarins Could Be Successfully Bioprospected from Fungi

Organisms such as cultivated microbes have provided much of the chemical diversity that has inspired syntheses and filled the pharmaceutical pipeline [36]. Bioactive natural products from fungi attract considerable attention from chemists and biologists [37]. Among these products, a significant place is given to the studies on the substances incorporating a coumarin moiety in their structure, owing to their antiviral, anti-HIV [38], antimicrobial, antitubercular, anti-inflammatory, cytotoxic, apoptotic, antiproliferative, anticancer [39], antiadipogenic, antidiabetic [40] activities of great pharmacological value. The principal medicinally important plant-derived coumarins are produced by fungal endophytes.

The term “endophyte” was introduced by De Bary in 1866 to define all microbes (including fungi, bacteria, cyanobacteria, and actinomycetes) that reside within plant tissue. Bacon and White have provided a conclusive and widely accepted definition of endophytes: Endophytes are microbes that colonize the living internal tissues of plants without causing any immediate overt negative effect [41]. Although the existence of symptomless fungi inside plant tissues has long been recognized, it is only recently that horizontally spread fungal endophytes have been studied in detail by mycologists, ecologists, and plant pathologists [42]. Horizontally transmitted cryptic fungal symbionts (endophytes of non-grass plants) are an ecological group of fungi, mostly ascomycetes, that reside in the aerial tissues and roots of plants without inducing any visual symptoms of their presence [1]. The main component of all definitions of endophytes was that the endophytes survive in living tissues of plants for a short or prolonged period without producing any visible symptoms. In the ecosystem, the main role fulfilled by these cryptically existing endophytes appears to be that of decomposers, as they are among the primary colonizers of dead plant tissues [43].

Reliable evidence has been obtained to suggest that at least a few endophytes have such roles as protecting plants against phytopathogens, enhancing the host plant’s tolerance to abiotic stresses [44], etc. Endophytes are capable of enhancing a host’s resistance against herbivores, insects, other pathogens, drought, variations in temperature and salinity, and heavy metal stress [45], since these fungi can biotransform inorganic and organic low-molecular-weight chemical agents along with macromolecules (heavy metal compounds, toxins, and other pollutants) [46]. Prolonged and sustained responsive mechanisms of endophytic fungus developed to withstand the host plant’s defense were proposed to act as selection pressure for establishing novel metabolic pathways [47].

The mounting interest in endophytic fungi as sources of biologically active compounds should be based on the thorough research on fungal relationships with host plants. The interaction between fungal endophytes and their host plants is characterized by a finely tuned equilibrium between fungal virulence and plant defense, in which disease symptoms may develop if the association is disturbed by either a weakening of plant defense or an increase in fungal virulence [48]. Moreover, endophytic fungi are thought to interact symbiotically with their host plant, so that the host plant provides nutrients to the endophyte, which in turn produces bioactive substances to enhance the growth and competitiveness of the host in nature. Indeed, many endophytes are apparently able to synthesize the same natural products that also occur in plants. It is nevertheless assumed that production of these respective compounds in planta does not proceed exclusively by endophytes but is rather the consequence of concomitant plant and fungal biosynthesis [49]. Endophytes have been shown to be a versatile source of many new biologically active chemical structures [18], and a rather large number of endophytic fungi are added every year to this ever growing list of biologically relevant strains producing valuable drug candidates, which is a testimony to the fact that only a tiny fraction of an estimated one million endophytic fungal species have been cultured and screened for their bioactivities [1].

**Figure 2:**
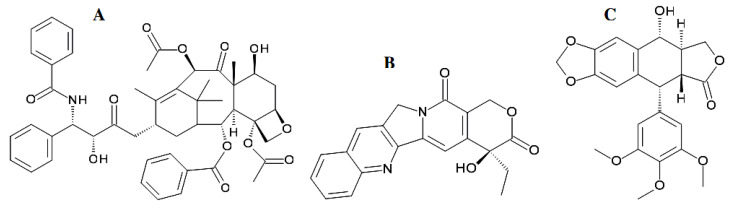
Compounds pioneered in studies unveiling the chemical potential of endophytic fungi: (**A**)—taxol (paclitaxel), (**B**)—camptothecin, (**C**)—podophyllotoxin.

Research into the chemical potential of endophytic fungi was first focused on the detection of pharmaceutically valuable plant constituents, e.g., taxol (paclitaxel), camptothecin, and podophyllotoxin (Figure 2), as products of fungal biosynthesis.

Endophytes did at first not receive much attention in the decades after the first discovery of this group of microorganisms [37]. This changed dramatically after the detection of paclitaxel (taxol) in the endophytic fungus *Taxomyces andreanae* that had been isolated from *Taxus brevifolia*, the latter being the original source of this important anti-cancer drug [50,51]. Biogenetic capacity for the production of some important drugs is far more widely spread in fungi than it is in plants. An example is just taxol, which was detected in other endophytic fungi that had been isolated from a wide range of host plants. In the years to follow, production of a target compound could be detected in endophytes from host plants that are not known to produce the given substance.

Another anticancer drug, cytotoxic plant alkaloid, camptothecin (Figure 2B) was initially isolated from the wood of *Camptotheca acuminata* [52] and identified in cultures of the endophyte *Entrophospora infrequens* isolated from *Nothapodytes foetida* with a maximum yield of 0.575 ± 0.031 and 4.96 ± 0.73 mg/100 g of dry cell mass in shake flasks and a bioreactor, respectively [52,53]. By now, camptothecin can be isolated from the endophytic fungus *Fusarium solani*, which is associated with the plant *Camptotheca acuminata* [54]. Podophyllotoxin (Figure 2C), a well-known anticancer agent, was generally found in *Podophyllum* sp., but due to the scarcity of this species, it was isolated from fungi such as *Trametes hirsuta* [55], *Phialocephala fortinii* [56], and *Fusarium oxysporum* [57]. Apoptotic studies with respect to *Alternaria* species as endophytes were carried out using podophyllotoxin from *Alternaria* sp. of *Sinopodophyllum hexandrum*, *Sabina vulgaris*, and *Sinopodophyllum emodi* [58,59,60]. Severe undesirable side-effects invoked by most anticancer drugs are the major hurdles limiting their application and therapeutic success. Therefore, the discovery of natural compounds with minimal toxicity in humans has been believed to be a major driving force for anticancer natural product investigations. The coupling of these factors has greatly facilitated efforts in the search for more effective and novel secondary metabolites as anticancer lead molecules. The plant extracts have traditionally been examined for these purposes. However, the endophytic fungi have also been revealed to biosynthesize the plant-associated key active agents [61,62,63].

Fungi are an under-studied niche in microbial drug discovery. Less than 20 percent of the described fungal species have been cultured and thoroughly investigated. These described species probably represent less than 5 percent of the total fungal species that await exploration [64,65]. Advantages for a microbial source of natural products are supported by several practical evidences. Fungi can be stored in a culture collection for a prolonged period of time, ensuring its availability. Fungi can be grown under the conditions of large-volume fermentation, thus producing a virtually inexhaustible supply of biomass or suspension to extract a target agent. Routine culture techniques could be modified by changing desirable parameters toward the enhanced productivity of these microbes to increase the yield in a dependable and reproducible manner [54]. That provides a more favorable response of fungi in respect to biosynthesis of the specific metabolites or lead compound analogues in the optimized synthetic route. Therefore, the important secondary metabolites from host plants and novel compounds with unpredictable architectures could be synthesized by fungi [66,67].

## 4. Fungi Is a Promising Source of Functional Coumarins

### 4.1. Fungal Coumarins with Anticancer Action

Inhibition of the proliferation of various cancer cell lines was observed for crude extracts from many fungi. The pharmacological significance of the fungus *Fusarium solani* was extensively explored by Kuriakose et al. [18]. The anticancer effect of ethyl acetate extract from a 21-day-old fungal culture was evaluated on five human cancer cell lines. Cytotoxic activity against all the tested human cancer cell lines was shown. Cancer cell apoptosis was induced by the *Fusarium solani* organic extract through the mitochondrial pathway [18]. The cytotoxic effect was found to be more profound in the human cervical carcinoma (HeLa) cells followed by the human ovarian carcinoma (OVCAR-3), the human breast adenocarcinoma (MCF-7), human prostate cancer (PC-3), and human hepatocarcinoma (HepG2).

The first report on the fungal coumarin isolated from an endophyte (*Alternaria* species), purified, characterized, and identified as a natural apoptotic agent appeared in 2015 [17]. Endophytic fungal coumarin from one of the *Alternaria* “species-1” isolated from the leaf part of *Crotalaria pallida* [68] was examined for in vitro apoptotic activity. The coumarin’s effect was tested against HeLa cervical cancer cell lines. These studies [17] found coumarin to be an effective agent in inducing apoptosis in HeLa cells, as well as capable of significantly inhibiting HeLa cell proliferation in a time- and concentration-dependent manner. Pentacyclic quinoline alkaloid camptothecin [54] and flavonolignan podophyllotoxin [56] have also been reported to be produced by endophytic fungi isolated from the associated host plants.

**Figure 3:**
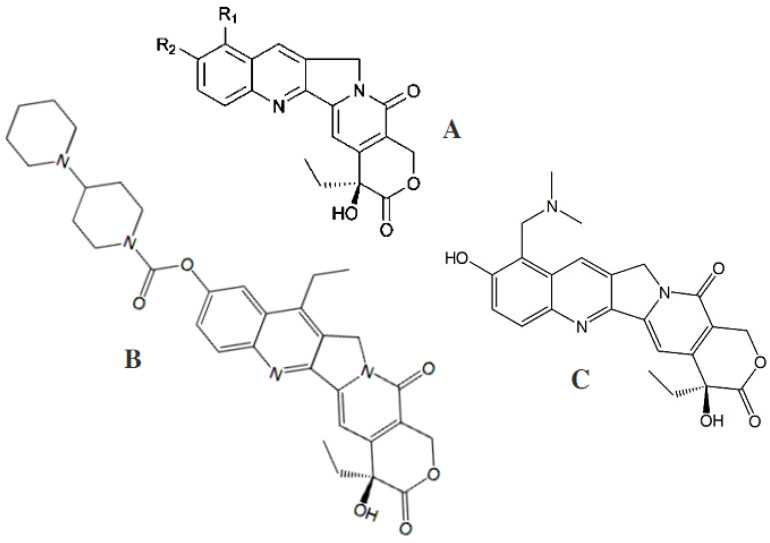
Structural formulas of camptothecin derivatives: (**A**)—9-methoxycamptothecin (R_1_=OCH_3_, R_2_=H) and 10-hydroxycamptothecin (R_1_=H, R_2_=OH); (**B**)—irinotecan; (**C**)—topotecan.

Camptothecin was initially isolated from the wood of *Camptotheca acuminata* (Nyssaceae), a plant native to mainland China. It was later isolated in 2005 using a fungal endophyte, *Entrophosphora infrequens*, isolated from the inner bark of *Nothapodytes foetida* [53], and somewhat later from a *C. acuminata* seed endophyte, *Neurospora crassa* [69]. In a year, camptothecin and two of its analogues, 9-methoxycamptothecin and 10-hydroxycamptothecin (Figure 3), were gained under fermentation conditions.

All three agents are produced by *Fusarium solani*, isolated from the inner bark of the medicinal plant *Camptotheca acuminate* [54]. For this purpose, the endophytic fungus *F. solani* was cultured in a rich mycological medium (Sabouraud dextrose broth) under shake-flask fermentation conditions. In addition, a parent complex alkaloid and its analogue, 9-methoxycamptothecin, have been isolated from the inner bark and from cell suspension cultures of *Nothapodytes foetida*, a small evergreen tree found in southern India and Sri Lanka that is unrelated to Camptotheca [70].

Podophyllotoxin (Figure 2C) is now being isolated from the endophytic fungus *Phialocephala fortinii* instead of its host plant *Podophyllum peltatum*, which was used in the past [56,57]. The endophytic fungal strains *Trametes hirsuta* and *Phialocephala fortinii*, isolated from *Podophyllum hexandrum* and *P. peltatum*, respectively, were reported to produce podophyllotoxin at a yield ranging from 0.5 to 189 μg/L [56]. Under the optimized fermentation conditions, a culture of the podophyllotoxin-producing endophytic fungus *Sinopodophyllum hexandrum* on the semi-synthetic medium biosynthesized up to 2.418 μg/L of podophyllotoxin in 6 days of fermentation duration [60]. Somewhat more recently, podophyllotoxin was also reported from *Fusarium oxysporum*, which is an endophyte of the medicinal plant *Juniperus recurva* that accumulates podophyllotoxin. The highest yield of podophyllotoxin produced by these endophytes amounted to 28 μg/g of dry mass [57]. This aryl tetralin lignan is highly valued as the precursor to clinically useful anticancer drugs. Substantial drug development of this compound class continues, including potential new uses for inflammatory diseases. Eyberger et al. [56] isolated two endophyte fungi, both strains of *Phialocephala fortinii*, from rhizomes of the plant *Podophyllum peltatum*. The fungi were identified through DNA sequencing and morphology. Both strains of fungi of *Phialocephala fortinii* are slow-growing and produce podophyllotoxin at low but measurable amounts in broth culture. The compound was confirmed through matching HPLC retention times, absorption spectra, and MS data to authentic podophyllotoxin. The yield of this compound ranged from 0.5 to 189 μg/L in 4 weeks of culture. These fungi have implications for the sustained production of podophyllotoxin independent of wild populations of the source plants. More recently, within the framework of previous podophyllotoxin research extension, an approach of bioactivity-guided fractionation was used by Pettit et al. [71] aimed at isolating the terrestrial plant *Bridelia ferruginea* cytotoxic constituents, among which the presence of the previously known podophyllotoxin derivatives was revealed, as isopicrodeoxypodophyllotoxin and deoxypodophyllotoxin found in the fungus *Aspergillus fumigatus* isolated from *Juniperus communis* [49]. In addition, those studies focused on structural modifications to yield 4-aza-podophyllotoxin. A number of the synthesized podophyllotoxin derivatives with alkyl and 4-fluorobenzyl substituents at the 4-aza position provided the greatest growth inhibitory activity (in a number of cases less than 0.03 ppm) against a panel of cancer cell lines [71].

**Figure 4:**
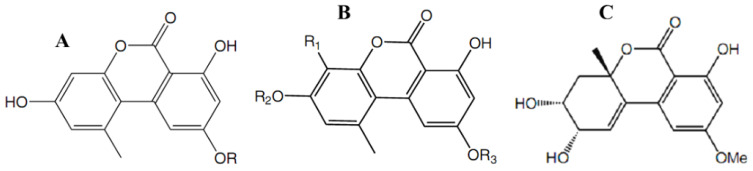
Structural formulas of alternariol derivatives: (**A**)—alternariol (R=H) and djalonensone, alternariol 5-O-methyl ether (R=Me); (**B**)—sulfated derivatives of alternariol (R_1_=H, R_2_=H, R_3_=SO_3_H) and alternariol monomethyl ether (R_1_=H, R_2_=SO_3_H, R_3_=CH_3_); 3′-hydroxyalternariol 5-O-methyl ether (R_1_=OH, R_2_=H, R_3_=CH_3_); (**C**)—4′-epialtenuene.

The fungal metabolite alternariol monomethyl ether (also known as djalonensone) (Figure 4A) was described for the first time from a plant source, *Anthocleista djalonensis* [72].

Later, djalonensone was reported from several *Alternaria* species (Pleosporaceae) residing in the traditional Egyptian medicinal plant *Polygonum senegalense* (Polygalaceae) [48]. External application of an extract obtained from fresh leaves of this plant is reported in folk medicine to be effective in treating skin troubles. Furthermore, species of *Polygonum* are known in traditional medicine for their diuretic, cholagic, antihemorrhagic, and antiseptic properties. Alternariol methyl ether isolated from the endophytic fungus *Alternaria alternata* showed anti-proliferative activity against human hepatocellular carcinoma cells (HUH-7). Along with this monoterpene diol with a chemical name tetrahydro-3-hydroxy hydroxymethylene-4-(3-hydroxymethylene prop-1-ene)-2H-pyran-2-one, another fungal metabolite of coumarin-nature, alternariol (Figure 4A), was isolated under laboratory conditions from endophytic strains of *Alternaria*. Alternariol was detected in the complex extracts from the corresponding host plants’ fractions using a liquid chromatography coupled with mass spectrometry technique [48,73].

The estrogenic potential of alternariol, as well as its inhibitory effect on cell proliferation by interfering with the cell cycle, was revealed by means of flow cytometry and microscopy of cultured mammalian cells [74]. Aly et al. [48] discovered later that chromatography-assisted analyses of these fungus extracts yielded several natural products. Chromatographic separation of an extract of the endophytic fungus *Alternaria* sp. isolated from the Egyptian medicinal plant *Polygonum senegalense* yielded 15 natural products, including four new compounds (Figure 4B,C), as well as two known ones (Figure 4A). Alternariol, its monomethyl ether, and a sulfated derivative of alternariol (Figure 4B) showed cytotoxic activity against L5178Y lymphoma cells with EC_50_ values ranging from 6.6–28.7 µM. When analyzed in vitro for their inhibitory potential against 24 different protein kinases, such compounds as alternariol, its monomethyl ether, a sulfated derivative of alternariol, and 3′-hydroxyalternariol 5-O-methyl ether (Figure 4A,B) inhibited several of these enzymes (IC_50_ values 0.6–36.4 µM). Not only djalonensone but also alternariol (Figure 4A) were identified as constituents of an extract derived from healthy leaves of the host plant *P. senegalense*, indicating the herbal natural product biosynthesis by the endophytic fungus within the host plant [48]. Extracts of the fungus *Alternaria* sp. grown either in liquid culture or on solid rice media exhibited cytotoxic activity when tested in vitro against L5178Y cells. Among the aforementioned 15 compounds, one-half showed cytotoxic activity, with EC_50_ values ranging from 1.7 to 7.8 µg/mL. When analyzed in vitro for their inhibitory potential against 24 different protein kinases, few compounds inhibited several enzymes (IC_50_ values 0.22−9.8 µg/mL). However, the substances comprising only one phenyl ring in their structure, 4′-epialtenuene (Figure 4C) and altenuene, its isomer by the 4’-position of hydroxyl radical, appeared to possess no cytotoxicity in the tests used [48].

The qualitative and quantitative characteristics of a pool of fungal secondary metabolites were dependent on the nutrient medium composition and density, with profound variations in a set of compounds being observed. From the extracts of *Alternaria* sp. grown in liquid Wickerham medium, new sulfated derivatives of alternariol and its monomethyl ether, as well as the known compounds alternariol and alternariol 5-O-methyl ether, were obtained [48]. When the fungus was grown on solid rice medium, a few new compounds of coumarin were identified, including 3′-hydroxyalternariol 5-O-methyl ether (Figure 4B), and altenuene. Furthermore, a new stereoisomer of altenuene was isolated from *Alternaria* sp. grown on rice medium in solid state, namely, 4′-epialtenuene (Figure 4C). Sulfated derivatives of alternariol, with a molecular formula of C_14_H_10_O_8_S gained from a mass spectrometric (HRESI/MS) technique, were isolated as reddish-white needles. Its UV spectrum showed considerable similarity to the UV spectra typical for alternariol derivatives. The carbon skeleton of alternariol, except for a few chemical shifts, changed and indicative of a sulfate substituent, or more precisely -O-SO_3_H, at a carbon atom C-5, was found. The molecular formula of another new substance, a sulfated derivative of monomethyl ether of alternariol, was determined to be C_15_H_12_O_8_S on the basis of the same method of [M + H]^+^ signal in the HRESI/MS. Spectroscopic and other data revealed the presence of the same carbon framework as in alternariol 5-O-methyl ether plus an -O-sulfate substituent at a carbon atom in the C-4’ position. The authors [48] were able to identify the latter substance as alternariol 5-O-methyl ether-4′-O-sulfate, a new natural product, as a result. Then the methanol (90%) soluble fraction from the fungal solid culture was further fractionated and purified by means of vacuum-liquid chromatography and preparative high-performance liquid chromatography, yielding the agents of coumarin nature, i.e., the new natural product 3′-hydroxyalternariol 5-O-methyl ether (Figure 4B), altenuene, and a stereoisomer of altenuene, 4′-epialtenuene (Figure 4C).

For the first time, the crystal structure of an alternariol methyl ether was obtained by a slow evaporation technique after isolation of an alternariol in the form of methyl ether from the secondary metabolites of the endophytic fungus *A. alternata* MGTMMP031 residing in the medicinal plant *Vitex negundo* [75]. This study was aimed at evaluating the anticancer activity of the crystallized compound alternariol methyl ether against hepatocellular carcinoma both in vitro and in vivo. Purification and characterization of the compound were performed, and the potential compound was identified as alternariol methyl ether. The crystal structure of this compound was unambiguously confirmed by X-ray analysis. Alternariol methyl ether has been examined for its anticancer and antibacterial properties. The assays showed its efficiency versus different bacteria and demonstrated marked anti-proliferative activity against the human hepatocellular carcinoma cells (HUH-7), both in vitro and in vivo. The mode of functionality involved cell cycle arrest, reducing the level of markers enzymes of liver cancer and preventing tumor growth [75]. The authors concluded that the alternariol methyl ether acts as a potential therapeutic target against hepatocellular carcinoma cells.

**Figure 5:**
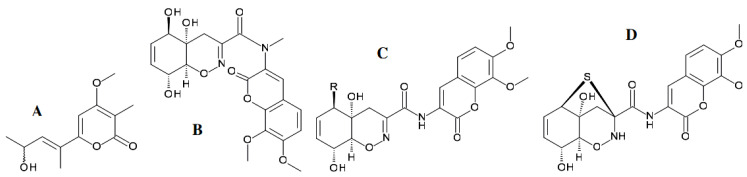
Structural formulas of trichodermamide derivatives: (**A**)—stemphypyrone, (**B**)—trichodermamide C, (**C**)—trichodermamide A (R=OH) and trichodermamide B (R=Cl), (**D**)—aspergillazine A.

The endophytic fungus *Stemphylium globuliferum* (Pleosporaceae) was isolated from stem tissues of the Moroccan medicinal plant *Mentha pulegium* (Lamiacae). Provided that *Stemphylium globuliferum* was grown on solid rice medium, the fungal extracts demonstrated considerable cytotoxicity when tested in vitro against L5178Y lymphoma cells. Chemical investigation and structural determinations on the basis of one- and two-dimensional NMR spectroscopy and mass spectrometry yielded new secondary metabolites, including a lactone stemphypyrone (Figure 5A). The isolated compound showed moderate cytotoxicity against L5178Y lymphoma cells.

Chemical investigation of the culture broth of the endophytic fungus *Eupenicillium* sp. (Trichocomaceae), isolated from the rainforest tree *Glochidion ferdinandi* (Euphorbiaceae) collected in Australia, afforded the new modified dipeptide trichodermamide C (Figure 5B). Its structure was established following the analysis of NMR, UV, IR, MS, and X-ray diffraction data, and it was assigned the molecular formula C_21_H_22_N_2_O_9_. Physicochemical data readily allowed the construction of a coumarin system substituted with methoxy groups at C-7′ and C-8′ and an *N*-methyl amide group attached to C-2′. The remaining protons in trichodermamide C were assigned to a tetraoxygenated cyclohexene system. The link between the coumarin and oxazine portions of trichodermamide C was also established. Thus, the structure contains the rare cyclic *O*-alkyl-oxime functionality and becomes only the fourth reported natural product that contains an 1,2-oxazine system [76].

Several natural products structurally related to trichodermamide C have been reported in the literature. Examples include trichodermamide A and trichodermamide B, distinct from each other by the radical (-OH or -Cl, respectively) at 5-position of the cyclohexene ring (Figure 5C). Both agents were isolated from cultures of the marine-derived fungus *Trichoderma virens* [77]. These two modified dipeptides possess a rare cyclic *O*-alkyl-oxime functionality incorporated into a six-membered ring, analogously to the above mentioned trichodermamide C. Another example of the natural products related to trichodermamide C (Figure 5B) is aspergillazine A (Figure 5D) from the terrestrial Australian fungal strain *Aspergillus unilateralis* MST-F8675. A complex set of metabolites detected by Capon et al. [78] included a highly modified novel heterocyclic dipeptide, aspergillazine A. The molecular structures of the compounds trichodermamides A, B, and aspergillazine A include a dimethoxylated coumarin system and a secondary amide functionality. Trichodermamide C was shown to display cytotoxicity towards the human colorectal carcinoma cell line HCT116 with an IC_50_ value of 0.68 μg/mL. Cytotoxicity assays were carried out using the A549 lung carcinoma cell line for comparison. The assay results demonstrated lesser activity of trichodermamide C versus the latter cell line expressed as an IC_50_ value of 4.28 μg/mL [76]. Examination for cytotoxicity with the cell line HCT116 revealed that only the chlorinated metabolite, trichodermamide B, but not the trichodermamides A, displayed significant in vitro cytotoxicity against the HCT116 human colon carcinoma cell line with an IC_50_ of 0.32 μg/mL [77].

**Figure 6:**
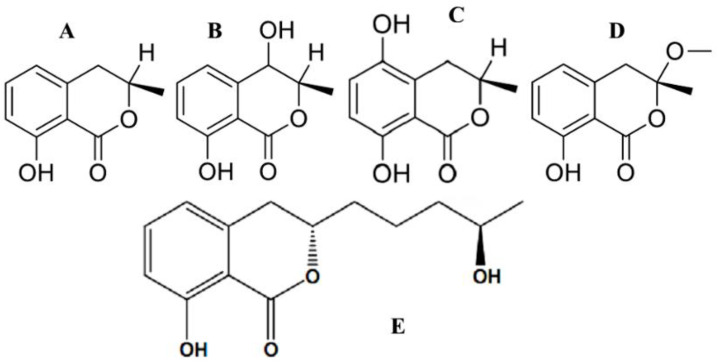
Structural formulas of mellein derivatives: (**A**)—mellein, (**B**)—*cis*-4-hydroxymellein, (**C**)—5-hydroxymellein, (**D**)—botryoisocoumarin A, (**E**)—aspergillumarin B.

A decade ago, Aly et al. [37] obtained an endophytic *Pestalotiopsis* sp. (Amphisphaeriaceae) from fresh healthy leaf material of *Rhizophora mucronata* (Rhizophoraceae) collected in the Dong Zhai Gang-Mangrove Garden on Hainan Island, China. Chemical investigation of this endophyte yielded new cytosporones J, K, and L, and new coumarins, pestalasins A–E. Principal component analysis of the fermentation culture of *Aspergillus flocculus*, an endophyte isolated from the stem of the Egyptian medicinal plant *Markhamia platycalyx* (Bignoniaceae), revealed a number of active metabolites. Under the optima-growth conditions, the 30-day rice culture provided the highest levels of the bioactive agents. The active fractions were purified further to yield seven metabolites, four of which were coumarin-structured, namely cis-4-hydroxymellein (Figure 6B), 5-hydroxymellein (Figure 6C), botryoisocoumarin A (Figure 6D), and mellein (Figure 6A), which inhibited the growth of chronic myelogenous leukemia cell line K562 at 30 µM [79].

Compounds which were mellein analogues share the same skeleton as aspergillumarin B (Figure 6E). Aspergillumarin B (C_14_H_18_O_4_) was reported by *Aspergillus* sp. for its moderate antimicrobial activity against *Staphylococcus albus* [80]. The coumarin 5,6-dihydro-6-pentyl-2*H*-pyran-2-one (C_10_H_16_O_2_) was isolated from *Simplicillium lamellicola* [81]. This substance, known as massoia lactone, was reported for its significant cytotoxicity against the MALME-3M human melanoma tumor cell line. Tawfike et al. [79] reported that massoia lactone was detected to be contributing to anticancer activity.

### 4.2. Acetylcholinesterase Inhibitory Activity of Coumarins

**Figure 7:**
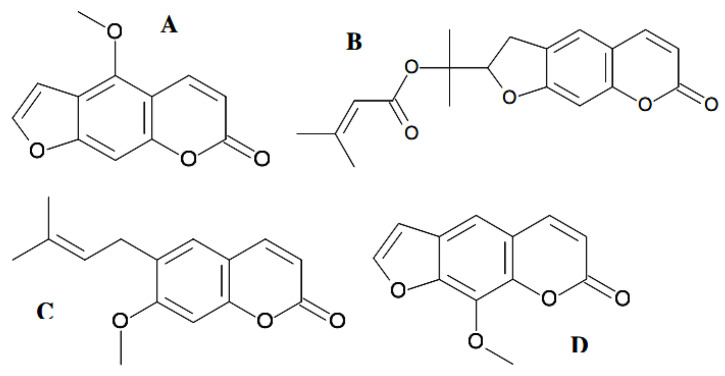
Structural formulas of acetylcholinesterase-inhibitory coumarins: (**A**)—bergapten, (**B**)—prantschimgin, (**C**)—suberosin, (**D**)—xanthotoxin.

Exploring small-molecular-mass inhibitors of the enzyme acetylcholinesterase to slow the breakdown of the neurotransmitter acetylcholine presents the mainstream direction for Alzheimer’s disease treatment [82]. The acetylcholinesterase inhibitory activity of the natural coumarins bergapten (Figure 7A), prantschimgin (Figure 7B), suberosin (Figure 7C), and xanthotoxin (Figure 7D) was also explored.

**Figure 8:**
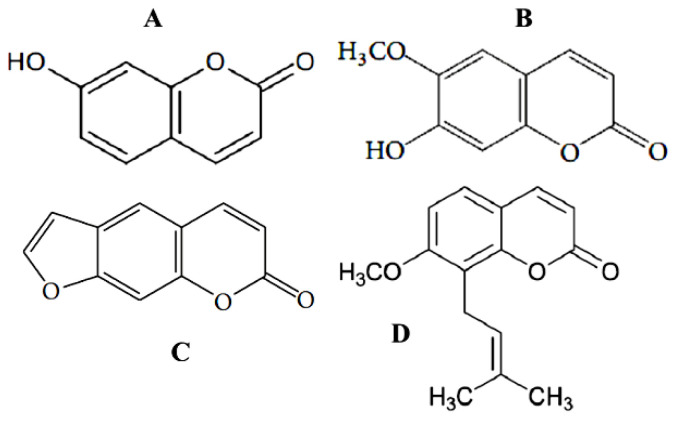
Structural formulas of antitumor and antiviral coumarins: (**A**)—umbelliferone, (**B**)—scopoletin, (**C**)—psoralen, (**D**)—osthole.

All coumarins inhibited the acetylcholinesterase enzyme, in which xanthotoxin showed the most inhibitory among them (IC_50_ = 39.64 µM). Another two naturally occurring coumarin derivatives with potent acetylcholinesterase inhibitory activity, scopoletin and umbelliferone, were isolated as components of a set of known substances from the mangrove endophytic fungus, *Penicillium* sp. ZH16, obtained from the South China Sea [83]. Umbelliferone (7-hydroxycoumarin) (Figure 8A) is a fluorescing compound and is used as a sunscreen agent.

It also shows antioxidant, anti-inflammatory, anti-hyperglycemic, antitumor, and antimicrobial activities. The anticancer activity of this agent against HepG2 cancer cells had not been reported until the research by Yu et al. [84]. Umbelliferone exhibits its anticancer effect via inducing apoptosis and cell cycle arrest. Later, to discover safer drugs, umbelliferone was used in the novel series of multifunctional hybrids containing a component called tacrine, the first acetylcholinesterase inhibitor introduced in therapy of Alzheimer’s disease, and a component with antioxidant functionality, coumarin [85]. A somewhat more complicated molecule, 6-methoxy-7-hydroxycoumarin, was named scopoletin (Figure 8B). This coumarin exhibits not only anti-acetylcholinesterase but also antitumor and antifungal properties. Scopoletin inhibits cancer cell proliferation by inducing apoptosis via reducing the protein content and diminishing acid phosphatase enzymatic activity [86,87].

Eighteen hybrids of coumarin–tacrine were synthesized by [82], the substitution being made at the C3 position of coumarin with the methylene chain as the spacer (Supplementary Table S1,**1**). Five to seven methylene groups were introduced in the structures. Coumarin components differ from each other by chemical substituents at C6 (–H or –OCH_3_ groups) and C7 (–H, –CH_3_, –OCH_3_ or –OCF3 groups). Some of the new dual-site inhibitors simultaneously interacted with multiple targets, like acetylcholinesterase and butylcholinesterase, and suppressed the formation of reactive hydroxyl radicals, thus alleviating the effects caused by the oxidative stress products. The structure with the simplest coumarin moiety (with –H at C6 and C7, see above) and six methylene groups was identified as the most potent dual-site acetylcholinesterase inhibitor, which was about two-fold higher potency than tacrine [82].

In Alzheimer’s disease treatment, multifunctional molecules have to be implemented to target not only the acetylcholinesterase system but also an extensive metal (copper, iron)-induced oxidative stress. That was accomplished using multifunctional tacrine-coumarin hybrids [88]. In this work [88], tacrine, 7-hydroxycoumarin, and tacrine-coumarin hybrids were examined. These particular compounds consisted of a tacrine unit and a 7-hydroxycoumarin substituted at the 4-position to yield a 7-hydroxy-2-oxo-2H-chromen-4-yl unit. Both units were linked by alkylenediamine or alkylene polyamine chains of different lengths via an amide functionality (Table S1,**2**) [88]. Free copper(II) ions have been recognized to occur in increased amounts in amyloid plaques and to catalyze the formation of free radicals (hydroxyl radicals, HO∙), mainly via the decomposition of hydrogen peroxide. The chelation of these ions by tacrine-coumarin hybrids prevents the Cu(II)-catalyzed decomposition of hydrogen peroxide, which results to a significantly suppressed formation of hydroxyl radicals and, as a result, reduces oxidative stress-induced damage. Therefore, the coumarin-containing hybrids under the conditions of redox-metals catalyzed oxidative stress may exert a protective effect important in the treatment of Alzheimer’s disease [88].

### 4.3. Antiviral Coumarin-Structured Agents

A lot of investigations referred to the inhibition impacts of diverse classes of natural coumarin phytochemicals on the functioning of viral proteins such as protease, integrase, reverse transcriptase as well as DNA polymerase, as well as preventing viral entry against a wide range of human viruses such as hepatitis B, hepatitis C virus (HCV) infections, infuenza, human immunodefciency virus (HIV), and herpes simplex virus (HSV) [16,19,33]. In two decades of extensive research, great progress has been achieved in the discovery of potent anti-HIV agents from nature. As earlier as two decades ago, substances with coumarin similar structures including saxalin, psoralen (Figure 8C), and its methoxy derivative bergapten (Figure 7A) were known to suppress HIV replication [89], and sometime later, such coumarins as mesoul and isomesoul were reported to prevent HIV replication in jurkat T cell [90]. A sesquiterpene coumarin kellerin; a natural furanocoumarin rutamarin, an aryl-coumarin glycycoumarin, and a simple coumarin osthole (Figure 8D) were reported to be anti-HSV and anti-HCV agents [91,92]. Moreover, other studies have reported that some of the natural coumarins such as xanthotoxin (Figure 7D), glycycoumarin, oxypeucedanin, pranferol, and heraclenol have anti-HIV activity [38,93]. Thus, several natural coumarins have been used as lead compounds because of their specific activity and low toxicity [94]. Many of them have the potential to interfere with particular viral targets, which can result in mechanisms of action complementary to those of existing antiviral drugs. Hymecromone, 4-methyl-7-hydroxycoumarin, served as a scaffold to be conjugated with several rather simple heterocyclic structures [95] to yield the antiviral agents. Bishnoi et al. [96] reported the synthesis of five hymecromone-based derivatives, in which antiviral activity was tested versus an RNA virus named Japanese encephalitis virus. Compounds displayed excellent antiviral activity with an inhibition percent of up to 100. A synthesis of seven coumarinyl-Schiff bases has been described by Mazzei et al. [97]. The antiviral activity of these conjugates was directed toward two phenotypes of the Hepatitis C virus, Bovine Viral Diarrhea Virus (BVDV), and Yellow Fever Virus (YFV). The results revealed that the synthesized coumarin hybrids have an encouraging antiviral activity versus the test phenotypes [97]. It should be noted that a 4,7-disubstituted coumarin skeleton likely favored the biological activity under study [98].

Natural compounds which possess lower side effects and toxicity have been used against the molecular targets of many viral proteins for inhibiting viral outbreaks [38]. Hence, they could be worthwhile candidates to fight against diverse viruses, including COVID-19. A pandemic viral disease caused by SARS-CoV-2 has generated serious damage for both the human population and the global economy [99]. Among the proposed drug targets within coronavirus, 3-chymotrypsin-like main protease (3CLpro) is an attractive candidate target for the inhibition of the viral replication cycle and the treatment of SARS-CoV-2 infections. 3CLpro of SARS-CoV-2 plays a critical role in the viral replication inside the host, or virulence, and is highly conserved across all known coronaviruses. As a result, the 3CLpro protein is a promising drug target for designing and developing effective antiviral drugs against SARS and other, possibly emerging coronaviruses. In their research, Abdizadeh et al. [99] evaluated some antiviral coumarin phytochemicals as potential inhibitors of coronaviruses’ 3CLpro by in silico approaches. The results demonstrated that the 3CLpro-glycycoumarin, 3CLpro-oxypeucedanin hydrate, and 3CLpro-inophyllum P complexes experienced fewer conformational fluctuations and were highly stable.

### 4.4. Efficacy of Coumarins against Bacterial and Fungal Infections

Natural coumarins as secondary-metabolites have appeared to show moderate-to-good potential as antimicrobial candidates [6]. Mellein (8-hydroxy-3(R)methyl-3,4-dihydroisocoumarin) (Figure 6A) got its name from a strain of *Aspergillus melleus*, which was the first known source of mellein [100]. It was reported from the endophytic fungal species *Septoria nodorum* in 1995 by Findlay et al. [101] followed by a large amount of similar research. The compound (R)-(−)-5-hydroxymellein (Figure 6C) was isolated for the first time from fungi of the genus Botryosphaeria in the first report on the isolation of -lactone derivatives from this genus [102]. Later, this compound was found in plants such as Moringa [103], and as the first isocoumarin reported in *Stevia* genus (Asteraceae) [104]. In the first report of the isolation of endophytic *Nigrospora* from the amedicinal plant *Moringa oleifera*, Zhao et al. [105] obtained mellein from the liquid nutrient media of the fungus *Nigrospora* sp. LLGLM003. The preparation exhibited antifungal activity against pathogenic *Botrytis cinerea* with an EC_50_ of 49.2 μg/mL. (–)-Mellein has been recognized for its activity against the protease of hepatitis C virus, antibacterial, fungicide, larvicide effects, whereas (+)-mellein is a potent phytotoxic, neurotoxic, and insecticidal agent that is remarkably effective against *Calliphora erytrocephala* [104]. The substances were obtained from the endophytic fungus *Arthrinium* state of *Apiospora montagnei*. The substances *R*-(−)-mellein and *cis*-(3*R*,4*R*)-4-hydroxymellein exhibited antimicrobial and antischistosomiasis effects, the latter against the parasites *Schistosoma mansoni* adult worms, with a 100% biocidal activity at the concentrations of 200 and 50 μg/mL, respectively [106].

Chemical analysis of the culture extract of the fungus *Nodulisporium* sp. (Xylariaceae), isolated from the plant *Erica arborea* (Ericaceae) growing on the Canary Islands, yielded six novel metabolites [107], including 5-hydroxy-2-hydroxymethyl-4*H*-chromen-4-one (Table S1,**3**). This substance, possessing a 4*H*-chromen-4-one structural subsystem, was tested for its antimicrobial properties using the agar diffusion assay in comparison to standard antibiotics. The assay showed antifungal and antialgal activities.

**Figure 9:**
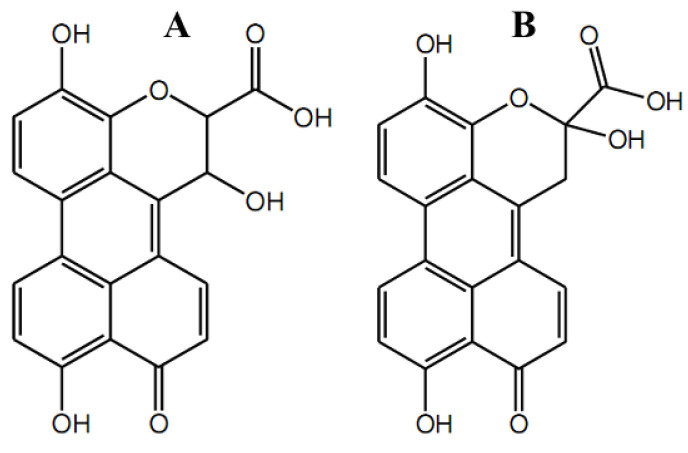
Structural formulas of xanalteric acids: (**A**)—xanalteric acid I and (**B**)—xanalteric acid II.

The fungus *Phomopsis* sp. (Valsaceae), isolated from the leaves of *Laurus azorica* (Lauraceae), a laurel tree from the Canary Island of Gomera, yielded three new metabolites [108]. All three (Table S1,**4**) were isocoumarins containing hydroxy groups in the molecule. The presence of a chloro-rine substituent in the structure of compound (Table S1,**4A**) distinguished it from two isomers (Table S1,**4B**,**C**). Cultures of the fungal endophyte *Ampelomyces* sp. (Leptosphaeriaceae), isolated from the medicinal plant *Urospermum picroides* (Asteraceae), collected in Egypt, yielded three new natural products [37], substituted lactone and two coumarins (Table S1,**5**). The latter two structures were hydroxypropyl dihydroxy isocoumarins, additionally containing -OH or two chlorine substituents in this hydroxypropyl radical at a pyrane ring, for (Table S1,**5A**) and (Table S1,**5B**), respectively. The extracts of the endophytic fungus *Alternaria* sp. (Pleosporaceae) isolated from fresh healthy leaves of the mangrove plant *Sonneratia alba* (Sonneratiaceae) collected in China, yielded two new compounds named xanalteric acids I (Figure 9A) and II (Figure 9B), along with eleven known metabolites [109].

**Figure 10:**
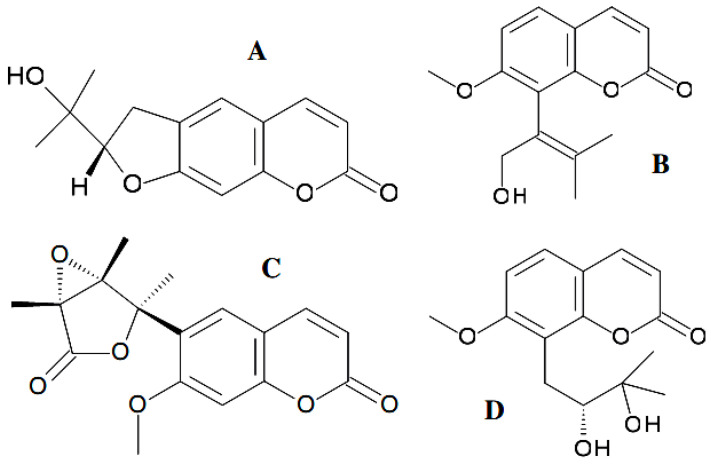
Structural formulas of antimicrobial coumarins: (**A**)—(+)-(S)-marmesin, (**B**)—murralonginol, (**C**)—micromelin, (**D**)—meranzin.

The metabolites were confirmed to be of fungal origin, and the structures of the natural products were unambiguously elucidated on the basis of extensive one- and two-dimensional NMR spectroscopic studies and mass spectrometric analysis. The structure of the xanalteric acids contained a carboxy group at a position adjacent to a pyrane oxygen instead of being a chromen-2-one system, while the xanalteric acids II were coumarin with an additional substituent, a carboxy group, at a position adjacent to a pyrane oxygen. These two new 10-oxo-10*H*-phenaleno[1,2,3-*de*]chromene-2-carboxylic acids (Figure 9) exhibited weak antibiotic activity against multidrug-resistant *Staphylococcus aureus* with MIC values of 343.40–686.81 µM [109]. Marmesin (Figure 10A) ((+)-marmesin belonging to furanocoumarins) was bioproduced by the endophytic *Fusarium* sp. ZZF41 that was isolated from the stem of the mangrove tree *Kandelia candel* from Dong Zai, Hainan, China [110].

Natural coumarin derivatives such as scopolin, osthenol, 6,7-dihydroxy-7-O-geranyl-coumarin (synonym is 7-O-geranyl-esculetin), dimethyl allyl psoralene, dimethyl allyl xanthyletin, and isoangenomalin exhibited antifungal activity [111,112]. Metal coordination plays an important role, since being coordinated with metals to yield metal complexes, coumarins demonstrated moderate-to-good antifungal efficacy versus many pathogenic fungi, including *Aspergillus flavus*, *Candida albicans*, *Trichophyton longifusus*, *Candida glaberata*, *Fusarium solani*, and *Microsporum canis* [113], as well as a rather high antibacterial activity against phytopathogens [114]. Coumarin derivatives were studied as a component of the basidiomycetes’ nutrient media. Bactericidal activity of the mycelial biomass products against phytopathogenic bacteria from *Micrococcus*, *Pectobacterium*, *Pseudomonas*, and *Xanthomonas* genera was found. The effect of nitro group-containing compounds as a component of the growth medium for mycelium, implemented in the potentially bactericidal specimen manufacturing process, appeared to be much more pronounced. Species-specific peculiarities of the above bioactivity manifestation for fungal biopolymers took place. Research into the ways of obtaining and the possibility of practical use of the biometals(II) complexes based on the coumarin fragment-containing systems seems to be due to the high biological activity of coumarins, which are quite promising for solving problems in crop production and plant protection. In a recent work, the complexes of Cu(II), Mn(II), and Zn(II) with 4-hydroxy-3-(3-oxo-1,3-diphenylpropyl)-chromen-2-one and 4-hydroxy-3-(3-oxo-1-(3-nitrophenyl)-3-phenylpropyl)-chrome-2-one were synthesized. The antibacterial activity of the products of these complexes biotransformation by basidiomycetes against a number of phytopathogenic bacteria has been determined. In respect to the extracellular fungal metabolites’ antibacterial activity, the leading place was occupied by the Cu(II) complex with 4-hydroxy-3-(3-oxo-1,3-diphenylpropyl)-chromen-2-one. Extracellular metabolites of *Laetiporus sulphureus* and *Lentinula edodes* facilitated the formation of biopreparations with more pronounced bactericidal activity [114].

Furocoumarin bergapten (Figure 7A) is 5-methoxypsoralen, a potential photosensitizing drug in the oral photochemotherapy of psoriasis. Bergapten, produced by the fungi *Alternaria brassicae*, *Botryodiplodia theobromae*, and *Penicillium* sp. [61,115], was reported to suppress pathogens’ growth, both bacterial and fungal, and to possess the best activity against *S*. *aureus* and *C. albicans*. This compound forms a stable combination with pyrimidine bases, causing DNA damage and the tumor suppressor gene Phosphatase and Tensin Homolog (PTEN)-mediated induced autophagy [21,116], indicating anticancer activity. For example, bergaPTEN induces metabolic reprogramming in human breast cancer cells [117]. Meranzin (Figure 10D) exhibits an antidepressant effect through regulation of the 2-adrenoceptor [118]. Meranzin, along with bergapten, is also found in grapefruit peels [20]. Both compounds are also produced by endophytic fungi, *Penicillium* sp., *Botryodiplodia theobromae*, and *Alternaria brassicae* [83,119]. *Botryodiplodia theobromae* Pat. belongs to the endophytic fungi that live within the tissues of medicinal plants and produce bioactive natural products. The endophyte was isolated from the leaves of *Dracaena draco* L. Ethyl acetate extract of *B. theobromae* with antibacterial activity was studied by means of LC–MS-based metabolite fingerprinting. Among 13 metabolites from various classes, coumarin and isocoumarins (bergapten, meranzin, and monocerin) were identified [119].

Naturally occurring coumarins, including fungal ones, possess a fair amount of moderate activity against *Mycobacteriun* strains. Many authors believed that the further modifications of these agents’ molecular structures would assist in accomplishing better performance as drug candidates. Chiang et al. elucidated the structures of 19 natural compounds by means of spectroscopic techniques (UV, IR, MS, ^1^H- and ^13^C-NMR, DEPT, COSY, NOESY, HSQC, HMBC, and MS analyses) [31]. Among the tested compounds, scopoletin (Figure 8B), isobavachalcone, and (*E*)-1-[2,4-dihydroxy-3-(3-methylbut-2-enyl)phenyl]-3-(2,2-dimethyl-8-hydroxy-2*H*-benzopyran-6-yl)prop-2-en-1-one exhibited the strongest antimycobacterial activities against *Mycobacterium tuberculosis* H_37_Rv, with MIC values of 42, 18, and 30 μg/mL, respectively. Scopoletin was also isolated within a group of seven known 7-oxygenated coumarins, along with two new 7-oxygenated coumarins, 7-demethylmurralonginol isovalerate and murralonginol [120]. All the found coumarin derivatives were tested against the *Mycobacterium tuberculosis* MtbH_37_ strain. The results testified to the inactivity of most compounds versus the *M. tuberculosis* strain, apart from murralonginol (Figure 10B) and micromelin (Figure 10C), which exhibited moderate activity (MIC 50 μg/mL) with reference to standard drugs.

## 5. On the Molecular Mechanisms Underlying the Pharmacological Effects of Fungal Coumarins

### 5.1. Grounds for the Apoptotic/Cytotoxic Activity of Coumarins

Fungi are known for their antitumor potential along with other medicinal applications. Cancer as a large group of diseases is rapidly becoming a pandemic all over the world, and in this understanding, humans are focused on searching for novel effective and economical therapeutic agents [70,121]. Cancer cells show an inherent ability to tolerate extreme conditions, such as those characterized by low nutrient and oxygen supply, by modulating their energy metabolism [122]. High toxicity is usually associated with chemotherapeutic drugs and their undesirable side effects, increasing the demand for novel antitumor drugs active against untreatable tumors, with fewer side effects, and/or with greater therapeutic efficiency [123].

Coumarin-derived compounds may have anticancer action through several ways, such as deactivation of the telomerase enzyme, inhibition of protein kinase activity, as well as suppression of oncogene transcription [124]. A tumor-suppressive effect can be achieved via reactive oxygen species (ROS)-induced oxidative stress in cancer cells [125]. To avoid blocking the free-radical-induced apoptosis of cancer cells, moderate pro-oxidants instead of large amounts of dietary antioxidants are preferable. Weak pro-oxidants are known to be capable of triggering the synthesis of endogenous antioxidants and thereby increasing the overall cellular antioxidant capacity for lowering various side-effects attendant to the drug-induced formation of ROS. Some coumarins have been found to act as mild pro-oxidants and might be appropriate candidates to accordingly boost the antioxidant capacity of cells [126]. All that together is in line with the opinion that, in general, polyphenols do not unambiguously perform as antioxidants in vivo [127], and that polyhydroxy flavonoids are known to increase the formation of hydroxyl radicals under Fenton reaction conditions [128]. Therefore, it seems not surprising that the hydroxycoumarin compounds containing multiple hydroxyl groups exhibit cytotoxic action by creating free radicals in cancerous cells, resulting in oxidative stress and apoptosis [129].

Several examples of typical compounds help to shed light on the molecular mechanisms underlying the pharmacological effects of fungal coumarins. Novobiocin (Table S1,**6**) was considered as a representative of a new class of potential anticancer chemotherapeutics. Novobiocin was found to act on eukaryotic cells by blocking the 90 kDa heat shock protein (Hsp90) [130]. Hsp90 as a molecular chaperone is critical for folding, stabilization, and activation of, among others, oncoproteins responsible for cancer progression. The C-terminus of Hsp90 was revealed to comprise a second ATP-binding pocket. The binding domain of novobiocin was localized in the C-terminal region of Hsp90, hence, the natural product novobiocin was the first C-terminal inhibitor discovered [131]. In C-terminal inhibitors, pro-survival responses are avoided. In contrast, in the known N-terminal inhibitors of this protein, some pro-survival signals are induced. Some of the novobiocin analogs exhibited a significantly enhanced anti-proliferative activity, the efficacy being assessed against several cancer cell lines [130]. In general, many of the proteins chaperoned by the heat shock protein Hsp90 (Hsp90 clients) are essential for the progression of various diseases, including cancer, Alzheimer’s disease, and other neurodegenerative diseases, as well as viral and bacterial infections [131]. Furthermore, novobiocin participates in a number of lysosome-based degradation pathways of cytosolic cargos by means of interaction with the members of the Atg8 family proteins as the key components of autophagy, in particular, with the human Atg8 family proteins, LC3A and LC3B. Novobiocin was very recently reported to be among the first nonpeptide inhibitors for these protein interaction targets [132].

Camptothecin (Figure 2B), a pentacyclic quinoline alkaloid incorporating a lactone chemical function, is a potent antineoplastic agent that exhibits strong antileukemic and antitumor effects in animals. Camptothecin inhibits topoisomerase I and, hence, exerts a cytotoxic effect by inhibiting the dissociation of the DNA–topoisomerase I complex during DNA replication. This drug penetrates vertebrate cells readily and targets topoisomerase I within minutes of exposure at a very low level, down to submicromolar concentrations. It does not bind to DNA or to topoisomerase I independently, but only to the complex formed by topoisomerase I when it cleaves DNA [133]. Examples of naturally occurring topoisomerase I inhibitors include semi-synthetic derivatives of camptothecin, such as irinotecan (Figure 3B) and topotecan (Figure 3C). 9-methoxycamptothecin and 10-hydroxycamptothecin (Figure 3A) are camptothecin analogues with similar potentials and no potential therapeutic drawbacks when compared to unmodified camptothecin. Both analogues are more water-soluble than camptothecin and more potent inhibitors of DNA topoisomerase I. Camptosar (irinotecan hydrochloride) has been approved for the treatment of colorectal carcinomas, and hycamtin (topotecan), the first orally available camptothecin derivative, and has been approved for the treatment of ovarian cancers and non-small-cell lung cancers [134].

**Figure 11:**
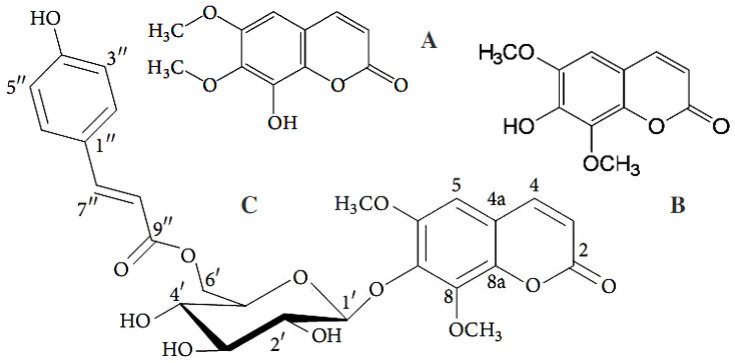
Structural formulas of fraxidin derivatives: (**A**)—fraxidin, (**B**)—isofraxidin, (**C**)—7-O-(6′-O-*p*-coumaroyl)-β-glucopyranoside isofraxidin.

Isofraxidin (7-hydroxy-6,8-dimethoxy coumarin) (Figure 11B) has shown a cytotoxic activity against cancer cells, an antihypertension activity, and anticancer, cardioprotective, and neuroprotective properties related to its unique pharmacokinetic profile and low levels of side effects [135].

Cyclic adenosine 3’,5’-monophosphate (AMP)—activated protein kinase α (AMPKα) is a key regulator of energy balance in many model species during hypoxia [136]. The investigation of the potential anti-inflammatory and immunomodulatory properties of different flavonoids, also classified as members of the benzopyrone group of secondary metabolites, on the production of pro-inflammatory interleukins and tumor necrosis factor α (TNF-α) in peripheral blood cells revealed that flavonoids have rather different effects [137]. Isofraxidin possesses anti-inflammatory activity by depleting, to a significant extent, infiltrating inflammatory cells and inflammatory cytokines [138]. Moreover, isofraxidin mainly regulates lipid metabolism and protects from related disorders by reducing triglyceride accumulation, TNF-α release, and the reactive oxygen species activation, enhancing the phosphorylation of AMPKα and acetyl coenzyme A carboxylase. Inhibition of lipogenesis is another property of isofraxidin, which is capable of reducing the hepatic expression of fatty acid synthase and 3-hydroxyl-3-methylglutaryl-CoA synthase 2. Compared to the parent compound isofraxidin, its naturally occurring derivative 7-O-(6′-O-*p*-coumaroyl)-β-glucopyranoside isofraxidin (Figure 11C) enhanced melanin synthesis by increasing tyrosinase activity and thus stimulating melanin accumulation [139]. This potent pigmentation enhancer performed its bioactivity without producing toxicity, as the use of the zebrafish vertebrate model demonstrated.

### 5.2. Coumarins Features Aiding in Neurodegenerative Disorders Therapy

According to several studies, many coumarins from natural or synthetic sources may be efficient for inhibiting acetylcholinesterase. Alzheimer’s disease is a neurodegenerative illness caused by a combination of factors associated, among others, with a significant loss of cholinergic neurons due to a deficiency of acetylcholine in certain brain areas [140]. That is attributed to altered activity of acetylcholinesterase, the enzyme that breaks down the neurotransmitter acetylcholine into choline and acetate. A number of substances with the ability to inhibit acetylcholinesterase could have a potential therapeutic effect [6]. Acetylcholinesterase inhibitors able to improve acetylcholine levels represent the vast majority of the currently approved drugs for Alzheimer’s disease therapy [141]. Tacrine (1,2,3,4-tetrahydroacridin-9-amine) (Supplementary Table S1,**7**) was the first approved cholinesterase inhibitor for the treatment of Alzheimer’s disease. A multifactorial origin of this disease involves a confirmed oxidative stress component. Hence, a series of new multifunctional hybrids containing tacrine and antioxidant functionalities have been prepared and evaluated [85,142], including a tacrine–coumarin hybrid [82]. All such hybrid molecules exhibit antioxidant and/or metal-chelating properties and are thus able to reduce the oxidative stress component in Alzheimer’s disease. For example, Hamulakova et al. recently demonstrated that when tacrine–7-hydroxycoumarin hybrids coordinate to free copper ions (typical for Alzheimer’s disease), Cu(II)-catalyzed decomposition of hydrogen peroxide via Fenton reaction is inhibited, which in turn may suppress oxidative stress-induced damage [88] (Table S1,**2**). Furthermore, cancer and this neurological disorder, Alzheimer’s disease, are believed to be diseases of opposite mechanisms characterized by enhanced resistance to cell death and premature cell death, respectively. However, a free radical-induced oxidative stress mechanism connects cancer and Alzheimer’s disease [125]. Therapeutic interventions of natural coumarins could modulate oxidative stress, since several coumarins have shown a well-expressed inhibitory effect on superoxide and hydroxyl radical generation [129].

Structure-activity relationship studies of a few coumarins were carried out with respect to their antibacterial and acetylcholinesterase inhibitory activities. The work by [143], in accordance with the known feasible “structure–activity relationships” among the coumarins, has demonstrated that the compounds possessing a methoxy group at the C5 carbon atom (e.g., bergapten), showed more potent antibacterial activity than the other coumarins. Furthermore, xanthotoxin with the OCH_3_ group at C8 had better acetylcholinesterase inhibitory activity as compared to other coumarins. Therefore, the methoxy group at C5 in furanocoumarins was suggested to be a functional site for antimicrobial activity, whereas this group at the C8 carbon atom could enhance acetylcholinesterase inhibitory activity. As it was exemplified by suberosin (Figure 7C), which was more active against *C*. *albicans* than against the bacteria, the absence of a furan ring in the molecular structure could be indicative of the more potent antifungal activity of coumarin. The quantitative and qualitative differences of the coumarin-nature compounds among plant species were considerable. It is important to find a species that has a wide range of antimicrobial activity in the genus [144].

### 5.3. Structural Prerequisites for Coumarins Being Antimicrobials

Antibiotics of the coumarin group are potential inhibitors of deoxyribonucleic acid (DNA) gyrase, a bacterial enzyme involved in cell division [130]. Research into the structure and function of the ATPase domain of DNA gyrase (bacterial topoisomerase IIA) has provided vital information for the understanding of the action of existing drugs and for the potential development of novel anti-bacterial agents. [145]. DNA topoisomerases are essential enzymes in all cell types and have been found to be valuable drug targets both for antibacterial and anti-cancer chemotherapy. Type II topoisomerases possess a binding site for ATP, which can be exploited as a target for chemo-therapeutic agents. The typical coumarin antibiotics inhibit the catalytic activity of DNA gyrase and topoisomerase IV (TopoIV) through inhibition of the ATPase activity of these heterodimeric type II topoisomerases [146], leading to inhibition of DNA synthesis and cell death. The use of Novobiocin (Albamycin) as an antibiotic for human therapy [147] could help shed light on the molecular mechanisms underlying the antimicrobial effect of coumarins. Novobiocin (Table S1,**6**), a fungus-derived aminocoumarin antibiotic, has been found to be effective in treating Gram-positive and Gram-negative bacterial infections [148]. This substituted coumarin is considered to be the first 4-hydroxycoumarin isolated as a microbial metabolite [149]. The Novobiocin structure consists of three distinct parts: a carbamoyl-methyl-lyxofuranoside (novobiose), a coumarin scaffold, and a benzamide side chain, which is a 3-prenylated 4-hydroxybenzoic acid moiety, and these parts are linked by glycosidic and amide bonds [131,150]. Thus, the phenolic 7-OH of the bicyclic aminocoumarin scaffold is L-noviosylated. The decorated noviosyl sugar is the pharmacophore that is presented by the planar aminocoumarin to the ATP binding site of the gyrase B subunit [151]. Two fungal aminocoumarin antibiotics, clorobiocin (Table S1,**8**) and coumermycin A1 (Table S1,**9**), structurally close to novobiocin, are effective at combating Gram-positive bacteria [152]. Ring A (3-dimethylallyl-4-hydroxybenzoic acid) is a structural moiety of novobiocin and clorobiocin [147]. The methyl group of coumarin and the carbamoyl group of novobiose are substituted by a chloro and a 5-methyl-pyrole-2-carbonyl group in chlorobiocin [146]. The dimeric structure of coumermycin A1 comprises a 3-methyl-pyrrolyl-2,4-dicarbonyl as a central core. All three coumarins mentioned possess the characteristic 3-amino-4,7-dihydroxycoumarin moiety which is also found in a number of other antibiotics. However, the distinct structures of the compounds result in differential sensitivity of bacterial cells to these antibiotic agents. The 2-NH_2_ group of the bicyclic aminocoumarin scaffold is ligated to a prenylated hydroxybenzoate unit (novobiocin and clorobiocin) or to a 5-methyl-2-pyrrolyldicarboxylic acid that links two aminocoumarin moieties (coumermycin). Coumermycin A1 appeared to be 50% percent more powerful an antibacterial agent than novobiocin in comparative studies revealing that coumermycin A1 caused greatly delayed DNA supercoiling of *Escherichia coli* [153,154].

A large family of broad-spectrum antibiotics involves fluoroquinolones. Some of them exhibited promising potency as new anti-tuberculosis agents. Fluoroquinolones consist of a 6-fluoro-4-quinolone-3-carboxylic acid core and a secondary amino group attached to the C7 position of the bicyclic ring juncture. Structurally similar compounds with an additional nitrogen atom at the 8-position (6-fluoro-1,8-naphthyridone-3-carboxylic acid core) were demonstrated to possess an antibacterial action profoundly influenced by the combination of substituents at the C3, C7, and N1 positions [155].

## 6. Multitarget Ligands of Functionalized Coumarins as Clinically Useful Drugs

### 6.1. Physicochemical Prerequisites and Toxicity

Some limitations of clinically approved compounds, particularly with regard to toxicity, lipophilicity, solubility, and development of resistance, are well known to interfere with their wider practical application. Particular physicochemical properties make a coumarin platform suitable for hybrid drug design. The aromatic, planar, and lipophilic nature of the 2*H*-chromen-2-one ring allows it to interact with a variety of counterparts, mainly lipophilic binding sites [156]. The occurrence of the benzopyrone platform in the structure of coumarins gives them a wide range of biological features. The lactone group allows coumarins to form strong or weaker polar interactions, e.g., hydrogen bonds, and to acylate protein targets [12]. Coumarin derivatives such as acenocoumarol, dicoumarolum, warfarin, hymecromone, carbochromen, and podophyllotoxin, to name a few, are approved for therapeutic purposes in clinics [14]. This successful usage of coumarin-based drugs in clinics has inspired further extensive research towards coumarin derivatives.

Secondary metabolites of coumarin chemical nature have been found to exhibit usually low toxicity associated with a variety of biological effects [157] and have raised considerable interest because of their potential beneficial effects on human health. Extensive research on the toxicological properties of coumarins has been carried out, and the important results from diverse toxicity studies have been collected in reviews [11]. Lacy and O’Kennedy reviewed the toxicity and carcinogenicity research conducted by the year 2004 and concluded that there was minimal health risk posed to humans from their exposure to food or cosmetic products naturally containing coumarins [158]. Conversely, there are papers that report on any type of significant toxicity of coumarin derivatives [11]. In general, the cytotoxic effects of coumarins are biological species-dependent, and consequently, different animal models cannot be used to evaluate the possible toxicity of coumarins in humans [159,160]. Moreover, coumarins, with their remarkable array of biological activities, act as a structural subunit of more complex natural products and are usually associated with low toxicity [161]. Recent human data-based studies revealed a tolerable coumarin dose intake of 0.1 mg/kg body, which must not be exceeded to avoid toxic effects [11].

A critical contributor to the pathogenic bacterium’s ability to evade the host immune system is its hydrophobic and complex cell wall, so incorporation of another lipophilic moiety, either as a substituent or a fused component into coumarin, alters the lipophilicity properties. For example, the lipophilicity of the anti-tubercular agents plays an important role in the penetration into mycobacterial cells [14], and increasing the lipophilic character of these compounds may also increase the anti-tuberculosis activity. Several strategies have been developed to overcome the pathogens’ resistance and improve the drug’s potential. One of the most practical approaches is molecular hybridization, which is based on the incorporation of two or more pharmacophores into a single molecule with a flexible linker.

### 6.2. The Series of Coumarin Hybrids with Important Class of Natural Products

A well-established approach to the design and synthesis of more potent drugs is the development of hybrid molecules through the combination of different pharmacophores in one molecular scaffold. This may lead to compounds with interesting biological profiles [157] and improved biological activity with respect to the corresponding lead compounds. Accordingly, by obtaining hybrids of coumarins with other pharmacophores, their biological activity could be enhanced [15]. Adopting this approach used before 2010 [161], several research groups have designed and synthesized hybrid molecules by coupling coumarins with a number of bioactive molecules like resveratrol, maleimide, and alpha-lipoic acid; these studies resulted in new compounds endowed with platelet antiaggregating, antioxidant, and anti-inflammatory activities. Fungal coumarin derivatives are implemented mainly in hybrid molecules with a few types of biological effects, namely anticancer and antitubercular activity. Among these properties, cytotoxic effects were most extensively examined.

#### 6.2.1. Anticancer Coumarin-Based Hybrids

Coumarin hybrid compounds can be considered as a promising approach to improve the pharmacological profile of the existing anticancer agents. New molecular tools thus obtained can deepen the knowledge of cancer progression and drug resistance mechanisms. A renewal of interest in coumarins in view of their flavonoid-like structure was noticed in the last decade for a deeper understanding of the molecular features needed to affect human cells [162]. Flavone- 5,6-dimethylxanthone acetic acid (DMXAA) emerged for its antitumor potential due to the induction of cytokines and consequent rapid haemorrhagic necrosis of murine tumor vasculature [162]. 3-Phenylcoumarin or 3-(4-fluorophenyl) coumarin structures substituted at the C8 position with a carboxymethyl group, showed a remarkable indirect toxic effect on tumor cells co-cultured with human MM6 cells, comparable to DMXAA despite the very low cytotoxicity observed. Moreover, the lack of cytotoxic effect for the unsubstituted 8-carboxymethyl coumarin highlighted the significance of the 3-phenyl ring on the core fragment [163], and the positive influence of fluorine compared to other substituents that led to inactive compounds.

A single molecule containing more than one pharmacophore, each with a different mode of action, could be beneficial for the treatment of cancer. Since a number of clinically useful anticancer drugs have genotoxic effects due to interaction with the amino groups of nucleic acids, chalcones may be devoid of this important side effect. Chalcones (1,3-diaryl-2-propen-1-ones) belonging to the flavonoid family constitute another important class of natural products. Due to the presence of strongly reactive α,β-unsaturated carbonyl groups, chalcones and their derivatives possess a wide spectrum of biological effects [164]. A series of coumarin–chalcone hybrids have been synthesized and shown to reveal a significant antitumor effect. Inspired by the earlier promising results, Sashidhara et al. [157] have designed a prototype and synthesized a series of novel compounds that have both coumarin and chalcone entities in one molecule and have evaluated them for their antitumor activity. The authors used chemical structures of some naturally occurring potent anticancer molecules that contained either coumarin or chalcone in a molecular structure. This series of coumarin–chalcone hybrids has been evaluated for their in vitro cytotoxicity against a panel of four human cancer cell lines and normal fibroblasts (NIH3T3). Among 21 compounds screened, the most promising one showed around 30-fold more selectivity towards cervical carcinoma cells C33A over normal fibroblast NIH3T3 cells with an IC_50_ value of 3.59 µM.

Cell division and progress of the cell cycle have been commonly recognized to be the most critical for unrestricted cancer cell proliferation [165], the disruption of the cell cycle being a major end goal of effective anti-cancer agents [166]. Three new indole-coumarin-thiadiazole derivatives were synthesized by utilizing a pharmacophoric hybridization approach. Selective caspase inhibition studies showed that indole-coumarin-thiadiazole hybrids [167] induced both extrinsic and intrinsic apoptosis in a caspase-dependent manner. These dual-acting compounds offer the possibility of a more comprehensive attack on cancer cells. The condensation reaction between 5-(1*H*-indol-3-ylalkyl)-1,3,4-thiadiazol-2-amines and 3-acetyl-6-bromo coumarin-2-one was carried out to yield three new thiadiazole hybrids incorporating bioactive coumarin and indole scaffolds. The structures of these thiadiazole hybrids, with spacers of varying lengths linking indole and thiadiazole units, were well-established using various spectroscopic techniques. 3-(1-(5-(3-(1*H*-indol-3-yl)propyl)-1,3,4-thiadiazol-2-ylimino)ethyl)-6-bromo-2*H*-chromen-2-one (IPTBC) exhibited dose-dependent cytotoxicity in breast adenocarcinoma (MCF-7) cells. IPTBC was thus identified as a new apoptotic and antimetastatic agent with significant differential toxicity towards MCF-7 cells. As for the plausible mechanism of apoptosis induction by IPTBC, the data presented from in vitro experiments [167] showed that hybrid IPTBC exhibited differential cytotoxicity in MCF-7 cells, and the cell kill was through an apoptotic mode. The results of that work imply that IPTBC can possibly act as a chemotherapeutic agent specific to breast cancer, both by the induction of apoptosis and by preventing metastasis.

The cancer chemopreventive properties of coumarins have been emphasized owing to the synthesis and the antitumor profile evaluation of a novel class of hybrid compounds obtained by introducing a substituted *trans*-vinylbenzene moiety onto a coumarin backbone. The stilbene structural skeleton consists of two aromatic rings bonded by an ethylene liker. Stilbene scaffold is a basic element for a number of biologically active natural and synthetic compounds and have become of particular interest owing to their wide range of biological activities. A successful design of novel stilbene-based hybrids in the fields of cancer, Alzheimer’s, and other relevant has been outlined [168].

Belluti et al. [161] explored the anticancer activities of stilbene–coumarin hybrid compounds, a novel class of conjugates obtained by the insertion of a properly substituted *trans*-vinylbenzene moiety on a coumarin backbone. The authors suggest that the 7-methoxycoumarin moiety combined with the 3,5-disubstitution pattern of the *trans*-vinylbenzene fragment are likely promising structural features to obtain excellent antitumor compounds endowed with an apoptosis-inducing capability. Xiao et al. [169] synthesized a series of 3-arylcoumarins structurally related to the parent natural trans-stilbenes. The new hybrids were tested for their antiproliferative effects against four human cancer cell lines. Compound bearing 7,8-diacetyloxy and 3′,5′-dimethoxy groups was the most active (IC50 value of 5.18 μM) against KB cell line.

Generally, the presence of a number of methoxy groups seems to be a fundamental requirement to obtain potent cytotoxic agents, and a 3,5-dimethoxyphenyl moiety in a number of resveratrol analogues was identified to play a pivotal role in conferring both the antitumor and proapoptotic activities [168]. In accordance with these observations, the presence of methoxy groups at specific positions, in both fragments of the hybrid molecules, was critical in conferring the antiproliferative activity. The 7-methoxycoumarin bearing a 3,5-dimethoxy *trans*-vinylbenzene function at the C4 position of the coumarin scaffold was found to be the derivative endowed with the higher antiproliferative activity on H460 lung carcinoma cells [161]. In addition, promising results were obtained by replacing the methoxyls at the 3 and 5 positions of the vinylbenzene moiety with methyl groups (–CH_3_), as evidenced by the effects of the compound characterized by a submicromolar IC_50_ value. The two just mentioned coumarin hybrids exhibited pharmacologically relevant antiproliferative activity and underwent deep biological investigations, thus revealing their ability to induce an appreciable level of apoptosis comparable to that of cisplatin, an extremely important but toxic drug [170].

Further efforts are being focused on the discovery of novel coumarin-based anti-breast cancer agents dual targeting the estrogen receptors (ER) and vascular endothelial growth factor receptor-2 (VEGFR-2). Estrogens are a group of steroid hormones with biological actions mediated through intracellular transcription factors called estrogen receptors, which belong to the nuclear receptor superfamily. ER mediates estrogen activity in many important physiological processes and plays a vital role in developing therapeutic agents destined for breast cancer treatment [171]. Estrogen receptor alpha (ERα) has provided an ideal pharmaceutical target, and a lot of ERα ligands have been developed as antagonists against ERα positive breast cancer. Selective estrogen receptor modulators (SERMs) are a special group of ligands that act as antagonists in breast tissue but as agonists in other tissues such as the cardiovascular system and bone [172]. Tumor angiogenesis is vital to cancer growth, metastasis, and invasion, and thus represents an attractive therapeutic target [173]. VEGFR-2 is a member of the receptor tyrosine kinase (RTK) family and the predominant effector of VEGF/VEGFR signaling in promoting angiogenesis in cancer [174]. Recently, several coumarin-type compounds were reported to block angiogenesis by inhibiting endothelial cell growth and have attracted considerable attention as antitumor agents [171]. In this context, the 3, 4-disubstituted-2H-chromen-2-one region, which contains a fused aromatic ring and a solvent side chain, has emerged as an intriguing scaffold for designing VEGFR inhibitors. Mohamed et al. (2019) [175] synthesized and tested for their anticancer potential in vitro, new thiazolylpyrazolyl coumarin derivatives targeting VEGFR-2 kinase and inducing cell cycle arrest and apoptosis. Some compounds showed potent cytotoxic effects against the human breast cancer cell line MCF-7 and VEGFR-2 inhibition. In addition, no noticeable toxicity was exhibited towards normal cells by HFB4. The hydrazide coumarin derivative, as the most cytotoxic compound in the research performed by Ahmed et al. (2020) [174], showed significant anti-VEGFR-2 activity, induced apoptosis, and activated caspase-9.

#### 6.2.2. Antitubercular Coumarin-Based Hybrids

Tuberculosis remains one of the most widespread and leading deadliest diseases [15] primarily caused by *Mycobacterium tuberculosis* (MTB) infection. The emergency of MTB’s new virulent forms as well as the co-infection between MTB and HIV has alarmed a global epidemic problem in tuberculosis control. Therefore, more potent drugs are demanded.

Fluoroquinolones have been proved to be a beneficial antibacterial class, safe in the low doses and short course, and currently represent a valuable class of drugs used to treat Gram-positive and Gram-negative bacterial infections [176]. Fluoroquinolones such as 4-quinolones are among the largest groups of chemotherapy agents used in clinical practice for the treatment of various bacterial infections. However, the development and spread of pathogens resistant to 4-quinolones makes them more and more ineffective. Thus, it’s imperative to develop new agents. Hybridization of other pharmacophores with 4-quinolone moiety has the potential to provide novel candidates with a synergistic effect in terms of efficacy, activity against resistant bacteria, and reduced toxicity in comparison to merely a mixture of the two drugs. Various 4-quinolone hybrids are under pre-clinical or clinical studies. The most emblematic examples were MCB3637, MCB3681, Ro-23-9424, and CBR-2092, which exhibited great in vitro and in vivo potency against both drug-sensitive and drug-resistant organisms [177].

However, fluoroquinolones are recommended as second-line agents, mainly in cases of resistance or intolerance to first-line anti-tubercular therapy. Other second-line anti-tubercular agents such as p-aminosalicylic acid, amikacin, cycloserine, capreomycin, and ethionamide are less effective and more toxic [14]. Even though a few antitubercular drugs have received approval for treatment, these are only recommended for MTB-infected patients without other treatment options because of the drug-associated side-effects. Therefore, hybrid molecules comprising fluoroquinolones/fluoronaphthyridones and coumarins were assessed for their anti-tubercular efficacy [178]. A series of gatifloxacin, ciprofloxacin, and 8-OMe ciprofloxacin coumarin hybrids were evaluated for their in vitro activities against *Mycobacterium smegmatis* CMCC 93202 and *Mycobacterium tuberculosis* H37Rv ATCC 27294. The results showed a remarkable improvement in lipophilicity as compared to the parent compounds. The activity of conjugate with the 7-methoxy-coumarin fragment bound via its C3 position to a gatifloxacin skeleton was found to be 2–8-fold more potent than ciprofloxacin, 8-OMe ciprofloxacin, moxifloxacin, and rifampicin. As for the latter substance, it should be noted that in the last 50 years, only a very few compounds have entered human trials after the discovery of rifampicin [15].

## 7. Methodology

### 7.1. Search Strategy

This review was undertaken by extracting the findings of the relevant works published in international electronic databases including ScienceDirect, Scopus, Google Scholar, PubMed, and Web of Science. The databases were thoroughly searched, and the relevant publication records were selected based on the keywords in accordance with the purpose of this study, as follows: natural compounds, coumarins, secondary metabolites, biosynthesis, fungi, endophytes, pathogenic bacteria, antimicrobials, anticancer activity, and fungal biotechnology. This review follows the framework of collected articles published between 2001 and 2022 in the form of original research articles, articles with their full text and data available, and studies that explore the pharmacological effects of coumarin in various types of diseases. Following a common search strategy [179], reference lists of included articles and relevant reviews were searched for any additional publications not captured.

Possible limitations of the review are the exclusion of grey literature sources [180] in the form of dissertations or theses, book chapters, conference proceedings, posters, or abstracts. The works of descriptive literature and studies conducted with secondary data were also excluded from the search. The fact that the scientific journals’ papers published in English were considered eligible for inclusion, could have the potential to miss relevant studies. The work did not perform a formal quality assessment of the selected papers.

### 7.2. Software Tools

For drawing 2D chemical structural formulas, a program called Biovia Draw 17.1 (Dessault Systèmes, Vélizy-Villacoublay, France) [181] was used. The original files in the working format of this program (“.skc”) were visualized by means of the structure editor tools [182], e.g., MDL ISIS/Draw (Elsevier MDL, San Leandro, CA, USA) or ChemDraw (CambridgeSoft, Cambridge, MA, USA) drawing packages [183]. The MDL Draw embedded object tool [184] was used to display chemical structural formulas as those incorporated into Microsoft Office documents, the latter being directly convertible into Portable Document Format documents with embedded molecular entities [185].

## 8. Concluding Remarks

Coumarin derivatives (coumarins, chromen-2-ones) attract interest because of, on one hand, their wide practical application and, on the other hand, the unique reactivity of the chromen-2-one system in molecular structure. The supply issue of coumarins raises a wide range of challenges in the discovery of new natural producers of these versatile agents. In the continuous search for novel drug sources, fungi have been proven to be a promising renewable reservoir of chemically diverse natural products. These microorganisms appeared to be capable of biosynthesizing the pharmaceutically precious natural coumarin-structured products, thus opening prospects of implementing the endophytic fungi in more rational approaches to alternative, ecologically safe, and sustainable sources of these products. The feasibility of wide-scale production of the fungal-derived medicinally important agents, including coumarin derivatives, must be proved in the near future.

## Data Availability

The data are contained within the article or Supplementary Material.

## Supplementary Materials

The following supporting information can be downloaded at: https://www.mdpi.com/article/10.3390/antibiotics11091156/s1, Table S1: Coumarin-derived structures used in this work.
