# Supplementary material for: Coumarins as Fungal Metabolites with Potential Medicinal Properties
Source: Antibiotics (Basel). 2022 Aug 26;11(9):1156. doi: 10.3390/antibiotics11091156 (PMC9495007; doi:10.3390/antibiotics11091156)
Supplement: Supplementary file 1 [file antibiotics-11-01156-s001.zip › antibiotics-1874753-supplementary.pdf]

Supplementary Table S1. Coumarin-derived structures used in this work

Code	Compounds' name	Compounds' structure	Reference
**1**	Tacrine–coumarin (C3-linked) hybrids (R1=H, CH_3, OCH_3 or OCF_3; R2=H or OCH_3 n=5, 6 or 7)	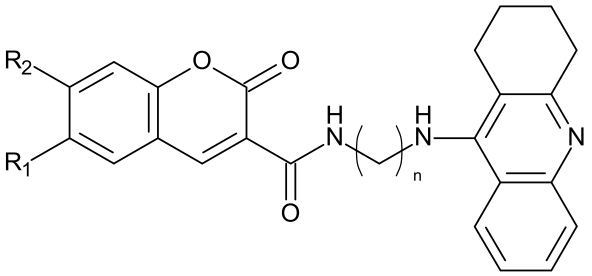	[82]
**2**	Tacrine–coumarin (C4-linked) hybrids	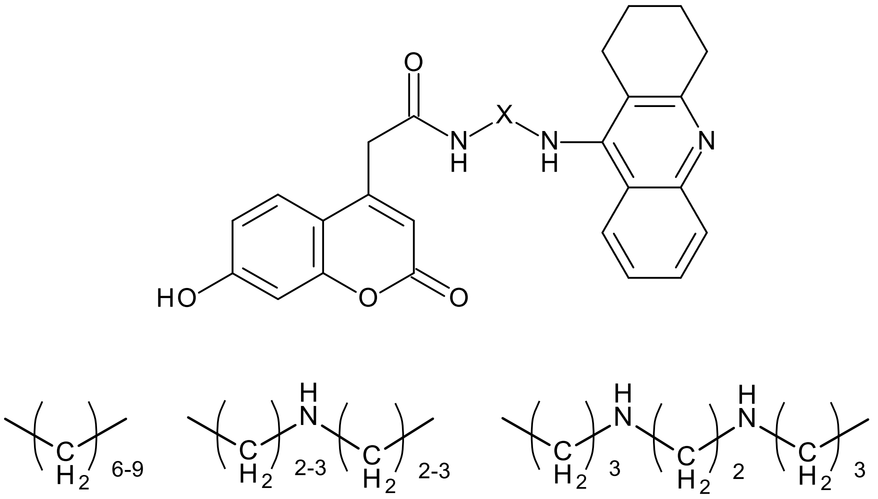	[88]
**3**	5-Hydroxy-2-hydroxymethyl-4*H*-chromen-4-one	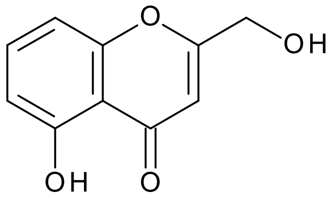	[107]
**4**	Antimicrobial coumarins from fungal endophyte *Phomopsis* sp. (**A**) (R_1=OH, R_2=Cl, R_3=H); (**B**) (R_1=OH, R_2= H, R_3=H); (**C**) (R_1=OH, R_2=H , R_3=CH_3)	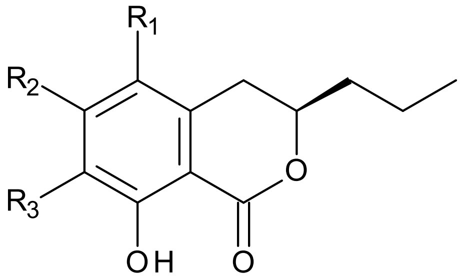	[108]
**5**	Dihydroxy isocoumarins from fungal endophyte *Ampelomyces* sp. (**A**) (R_1=OH, R_2=H); (**B**) (R_1=Cl, R_2= Cl)	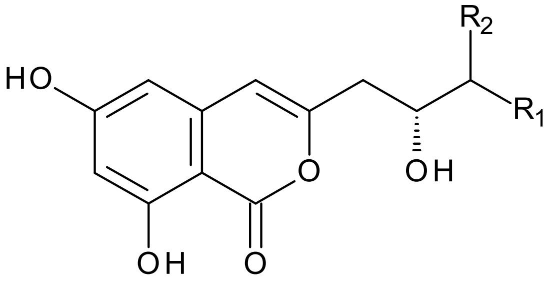	[37]

**6**	Novobiocin	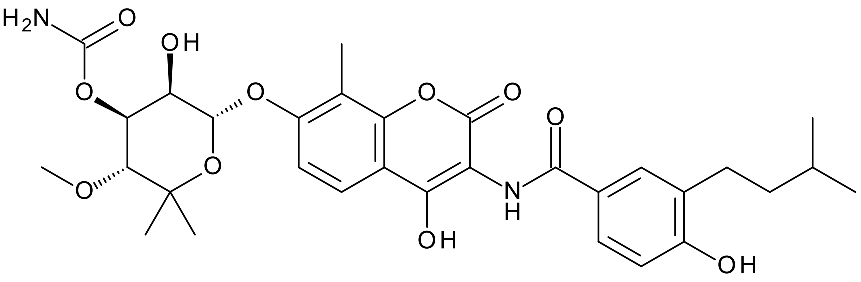	[130]
**7**	Tacrine	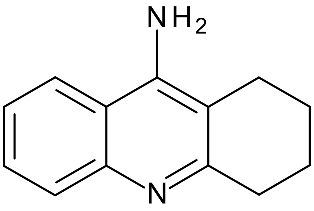	[142]
**8**	Clorobiocin	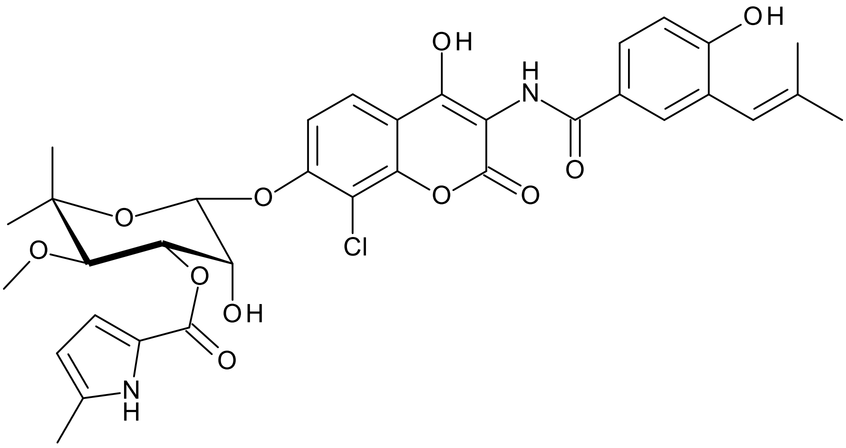	[147]
**9**	Coumermycin A1	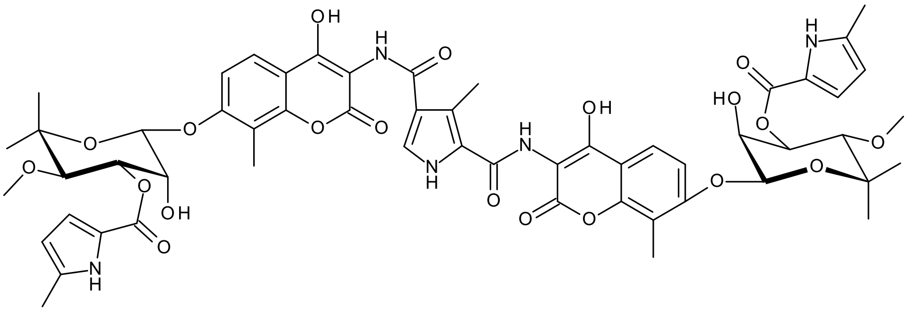	[146]
